# Influence of heat-assisted vat photopolymerization on the physical and mechanical characteristics of dental 3D printing resins

**DOI:** 10.1038/s41598-025-85529-7

**Published:** 2025-01-11

**Authors:** Jung-Hwa Lim, Seung-Ho Shin, Young-Eun Jung, Hongseok An, Jong-Eun Kim

**Affiliations:** 1 Department of Prosthodontics, Yonsei University College of Dentistry, Yonsei-ro 50-1, Seodaemun-gu, Seoul, 03722 Republic of Korea; 2 Department of Orthodontics, New York University College of Dentistry, 345 E 24th St, New York, NY 10010 USA; 3 Department of Oral Rehabilitation and Biosciences, Oregon Health & Science University School of Dentistry, 2730 S Moody Ave, Portland, OR 97201 USA

**Keywords:** Vat photopolymerization, 3D-printed crown, Hot lithography, Mechanical properties, Biomedical resins, Fixed prosthodontics, Dental biomaterials

## Abstract

The effects of heat-assisted vat photopolymerization (HVPP) on the physical and mechanical properties of 3D-printed dental resins, including the morphometric stability of 3D-printed crowns, were investigated. A resin tank was designed to maintain the resin at 30, 40, and 50 ℃ during the 3D printing process. Test specimens were fabricated using a commercial dental resin, with untreated resin serving as the control group. Key properties such as viscosity, curing kinetics, surface microhardness, flexural properties, and dimensional accuracy were evaluated. The viscosity of the resin decreased significantly (*P <* 0.05) with increasing temperature, thereby enhancing its flow properties. Photo-DSC analysis revealed a 17.58% increase in peak heat flow at 50 ℃, indicating accelerated polymerization. Surface microhardness improved significantly (*P <* 0.05) with HVPP, though a slight reduction was observed at 50 ℃ compared to that at 30 and 40 ℃. The flexural strength, modulus, and resilience were significantly enhanced (*P <* 0.05) at higher temperatures, with 50 ℃ yielding the best mechanical properties. However, 3D morphometric analysis showed increased root mean square deviation from the CAD design at elevated temperatures. Our results suggest that HVPP enhances the durability of dental prostheses, although careful optimization of the printing temperature is essential to balance their strength and accuracy.

## Introduction

Vat photopolymerization (VPP) employs liquid photopolymer resins selectively cured by light in a layer-based process and is particularly favored owing to its rapid curing reactions and the high resolution of optical sources^1^. The high resolution and favorable processability of this technique have led to its rapid adoption in dentistry^2,3^. In prosthetic dentistry, conventional photopolymer resins are primarily composed of (meth)acrylates and light-sensitive photoinitiators that initiate polymerization by generating free radicals^3^.

Typically, 10–20% conversion of dimethacrylates is sufficient for gelation, defining the shape of prostheses during 3D printing^4,5^. However, the 3D-printed part is unsuitable for clinical use because of the presence of the remaining uncured resin fractions of the diluent, photoinitiators, and other monomeric components^6^. Hence, to prevent biological toxicity and degradation, all 3D-printed parts must undergo thorough cleaning and post-curing^7,8^. Although 3D printing offers streamlined workflows, standardization of the technical sensitivity of the post-processing stage remains a challenge. Furthermore, the rapid development of dental 3D printing resin materials has led to increased variations in photopolymers, including differences in filler loading percentages, transmissibility, viscosity, and monomer–oligomer combinations. Therefore, the effects of solvents and temperature during cleaning and post-curing must be carefully managed based on the thermomechanical properties of each material to avoid distortion of the printed dental prostheses^9,10^.

Given the variability introduced during the post-processing stage, an alternative approach that reduces the dependence on these factors could promote the growth and general adaptation of dental 3D printing polymers. Conventional methacrylate polymers used in dentistry rely on a heat-polymerized mechanism, in which the heat source ensures stable conditions for radical generation, achieves a higher degree of polymerization, and strongly influences bulk curing^11^. Recent studies have proposed heat-assisted vat photopolymerization (HVPP), a recent advancement in light-induced technology that enables the minimization of solvent or diluent in the resin formulation composition^12–14^. The HVPP technique involves the introduction of a heated printing chamber that can enable printing at elevated temperatures, useful for studying the effect of temperature on the printing process^15–17^. Additionally, it improves UV-curing kinetics by accelerating the process with thermal energy, leading to a higher degree of conversion^14^.

Although optimizing curing kinetics can enhance polymerization and improve physical properties and biocompatibility, the success of additively manufactured dental prostheses is predominantly influenced by their morphological reproducibility^18^. Furthermore, the 3D printing resins currently used in clinical practice are primarily methacrylate-based, which are sensitive to stresses during polymerization, leading to a loss of dimensional accuracy^4^. Previously reported HVPP findings and methods employ markedly higher temperatures than those suitable for dental 3D printing resin formulations^19^. Given that 3D printing resins approved for clinical use have a narrow range of transition temperatures, HVPP would warrant specific optimization. However, this research area remains minimally explored, with limited evidence available in the literature specifically addressing the 3D printing of dental prostheses. Therefore, in this study, we aimed to investigate the effects of HVPP and identify the optimal temperature range for 3D printing resins used in clinical dentistry. For this, a customized resin tank was fabricated to control the temperature during printing, which was evaluated in the range of 30 to 50 ℃. The null hypothesis was that 3D printing at increased resin temperatures has no significant effect on the physical properties or accuracy of the printed objects.

## Results

### Viscosity

**Fig. 1:**
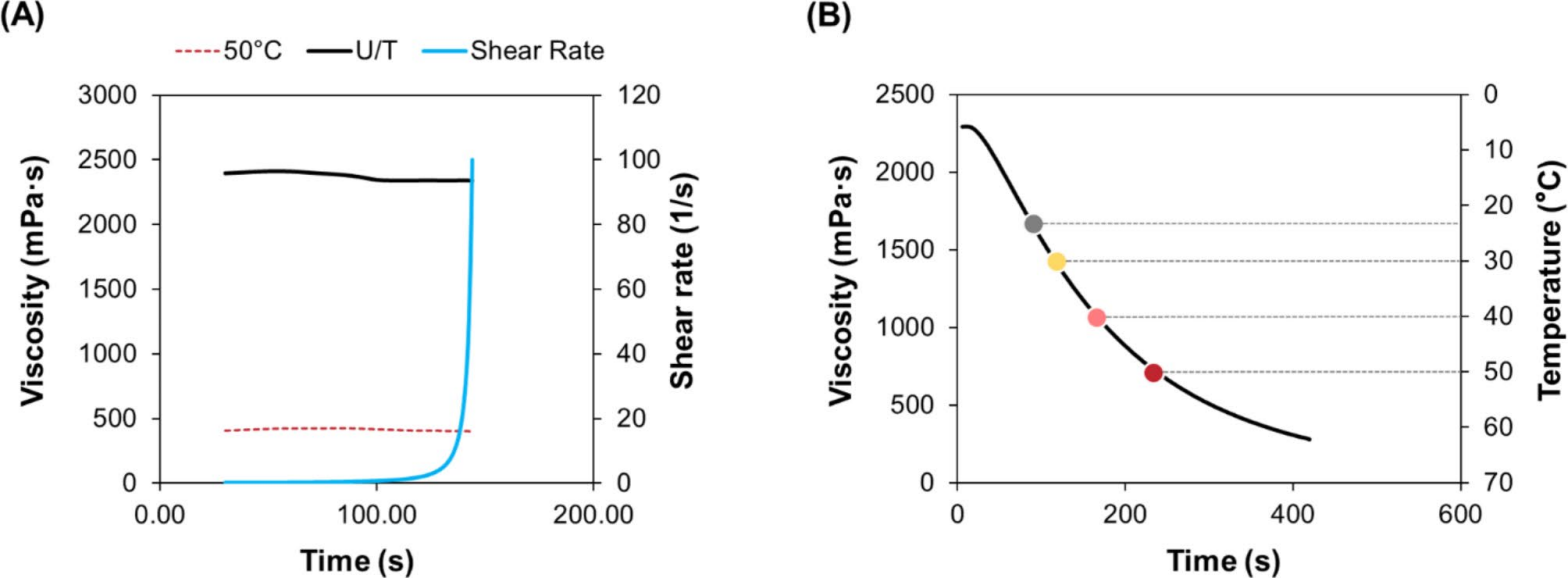
Characterization of the temperature dependence of the viscosity of the resin. Difference in the viscosity of the resin (**A**) before and after heating, and (**B**) real-time change in viscosity under constant heating (5.01 ℃/min).

The resin viscosity showed marked differences between the untreated resin (U/T, 2355.28 mPa.S) and the resin pre-heated to 50 ℃ (10–11 ℃/min, 410.32 mPa.S), as shown in Fig. 1A. Under a constant temperature increase of 5.01 ℃/min, the viscosity changes were 2294.9, 1829.9, 924.86, and 494.41 mPa.s for the U/T, 30 ℃, 40 ℃, and 50 ℃ groups, respectively (Fig. 1B). These results confirmed that the heating rate had a significant effect.

### Photo-DSC analysis of the curing behavior of the 3D printing resin

**Fig. 2:**
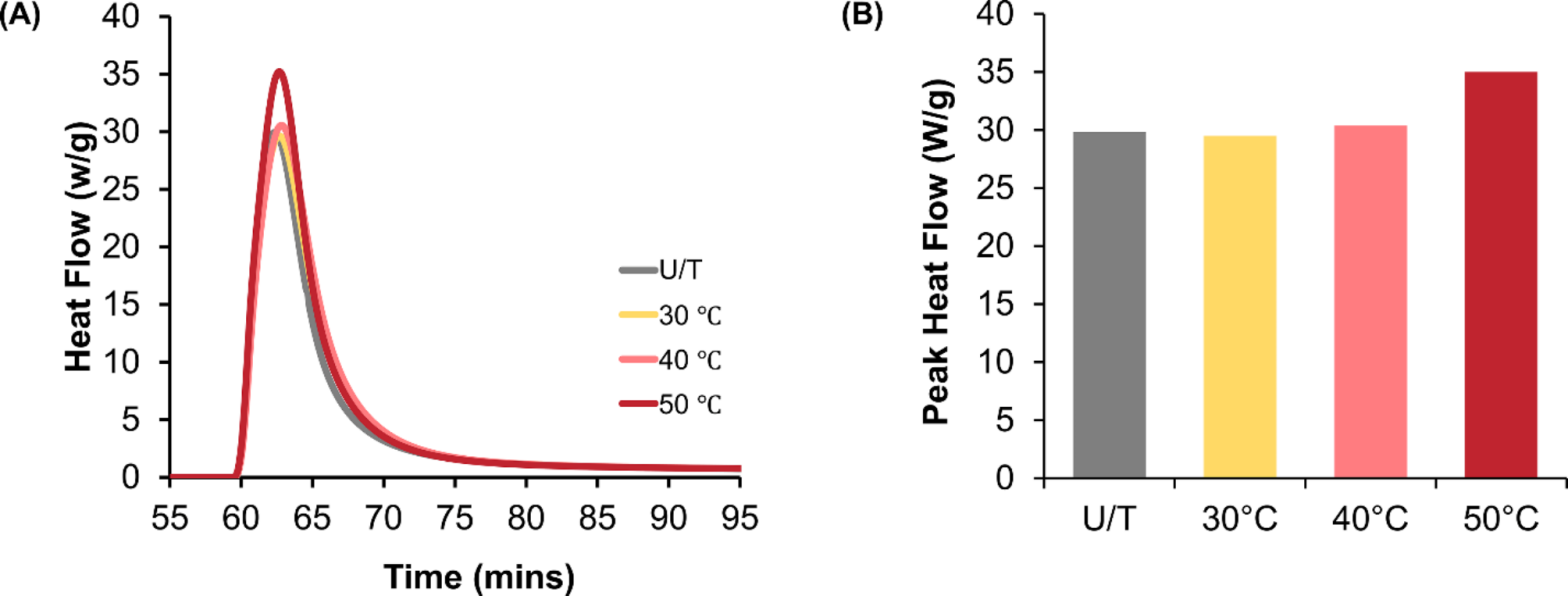
Photo-DSC results showing (**A**) comparison of the heat flow observed with tests performed at the target temperatures. (**B**) Comparison of the peak heat flow values between the groups.

Analysis of the photo-DSC results for the dental resin, with tests initiated at different starting temperatures, showed differences in their curing behavior (Fig. 2A). The observed peak heat flow values were 29.49 W/g at 30 ℃, 30.37 W/g at 40 ℃, and 35.00 W/g at 50 ℃. The U/T reference group exhibited a peak heat flow of 29.82 W/g. A 17.58% increase in peak heat flow was observed when the initial polymerization temperature was 50 ℃ (Fig. 2B).

### Surface microhardness

**Fig. 3:**
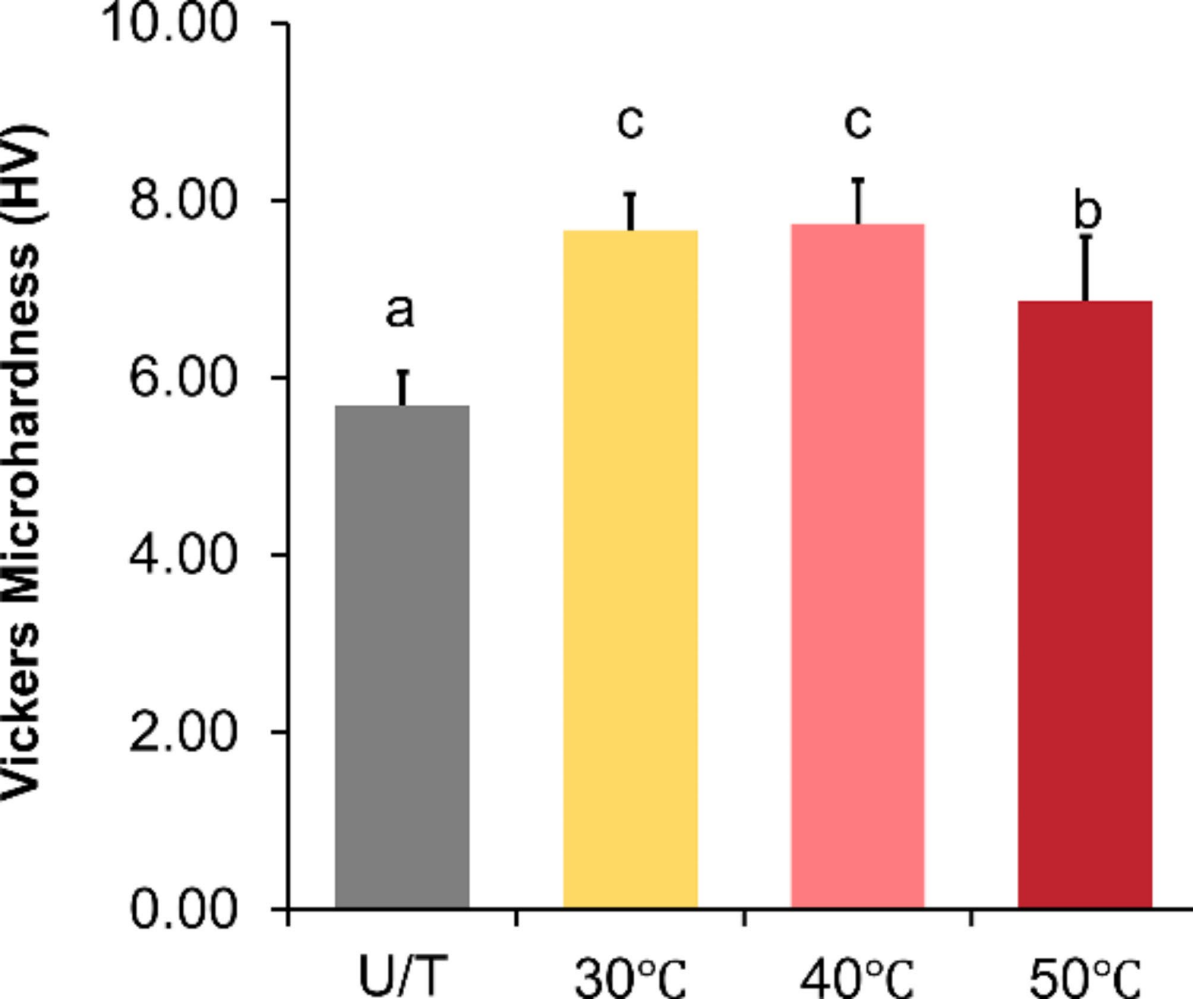
Vickers surface microhardness comparison between the tested groups. The different lower-case letters indicate significant differences (*P <* 0.05) between the groups.

U/T (5.68 ± 0.38 HV) exhibited the lowest microhardness followed by the 50 ℃ (6.86 ± 0.73 HV), 40 ℃ (7.74 ± 0.49 HV), and 30 ℃ (7.66 ± 0.4 HV) specimens. The microhardness of the U/T group was significantly different (*P <* 0.001) compared to the HVPP groups. Among the HVPP groups, the microhardness of the 50 ℃ specimens was significantly lower (*P <* 0.05) compared to those of the 30 ℃ and 40 ℃ groups (Fig. 3).

### Flexural strength and modulus

**Fig. 4:**
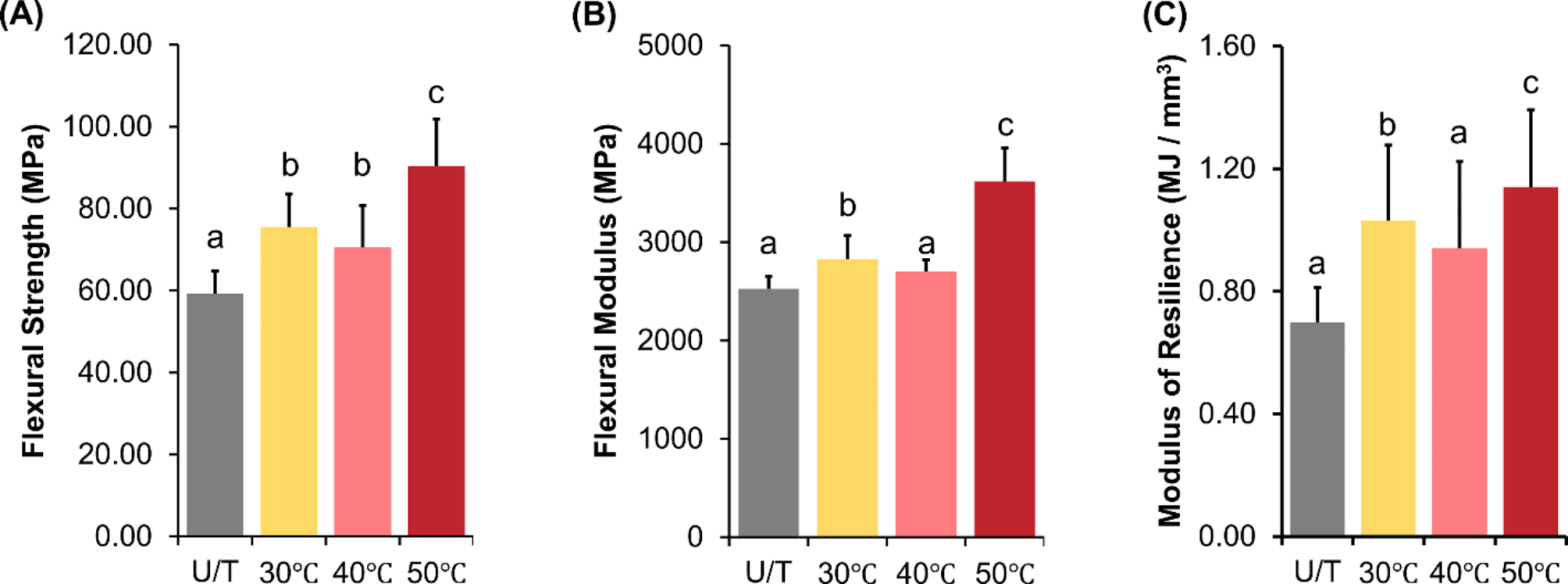
Comparison of the (**A**) flexural strength, (**B**) flexural modulus, and (**C**) modulus of resilience between the groups. The different lower-case letters indicate significant differences (*P <* 0.05) between the groups.

Comparison of the flexural behavior under the three-point bending test showed a statistically significant difference (*P* < 0.05) between the groups, with the highest values observed for the 50 ℃ group (Supporting Table S1). Specifically, the flexural strength increased significantly in the following order: U/T < 40 ℃ < 30 ℃ < 50 ℃ (Fig. 4A). Similar trends were observed for the flexural modulus (Fig. 4B) and modulus of resilience (Fig. 4C).

### Printing layer topography

**Fig. 5:**
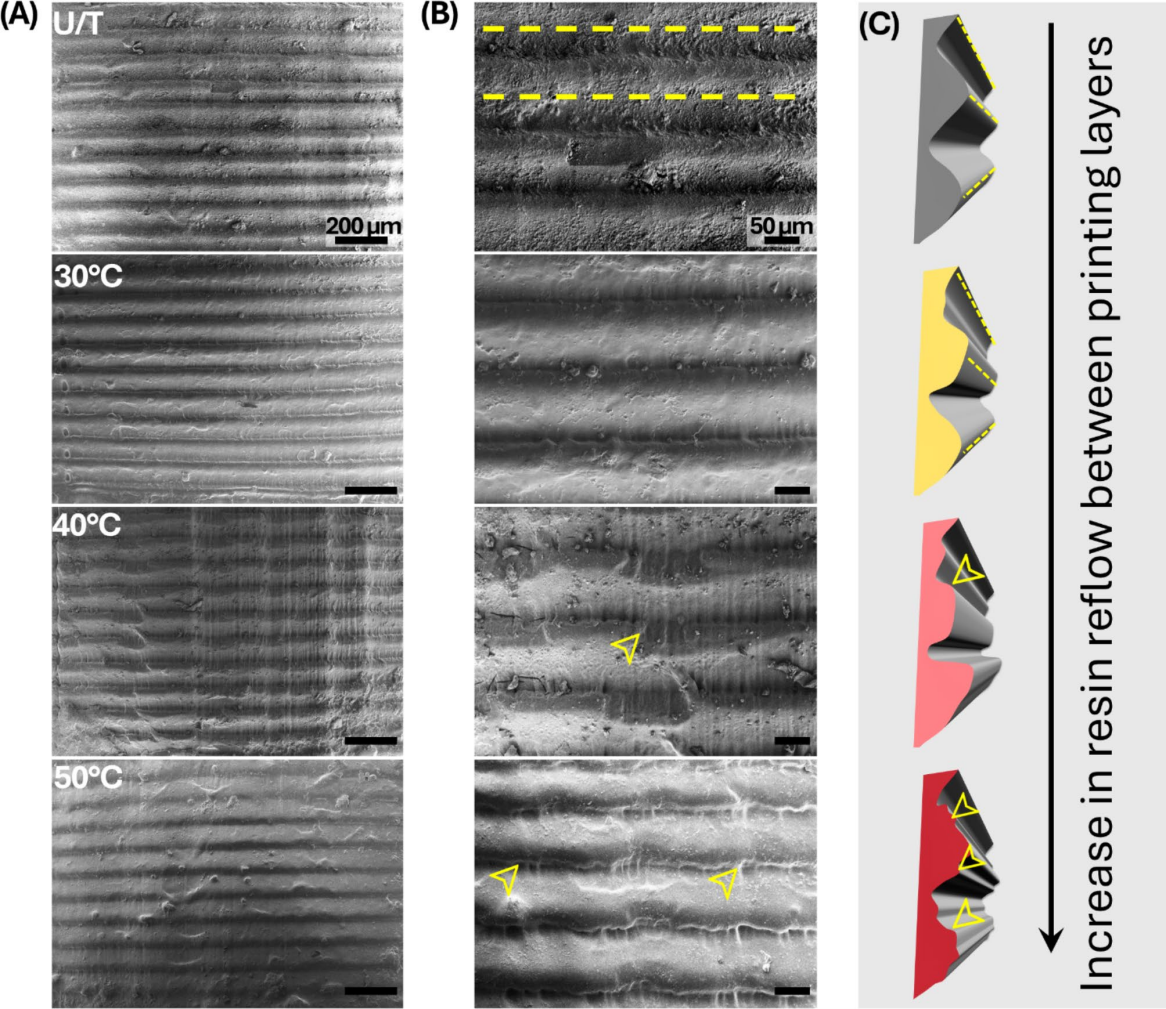
Scanning electron microscopy images of the 3D-printed samples. Comparison of the printer layer intervals (dotted-yellow lines) and their definition at (**A**) 90× (scale: 200 μm) and (**B**) 250× (scale: 50 μm). (**C**) Schematic of the observed changes in the layer definition marked by region of reflow (yellow arrows).

The scanning electron microscopy (SEM) images of the interlayer region of the 3D-printed samples showed stable and continuous structures in all groups (Fig. 5). However, the boundary definitions between the layers exhibited notable variations. Specifically, U/T specimen exhibited the initial state of the 3D-printed surface with distinct horizontal lines, which is characteristic of the layer-by-layer deposition process. Relative uniformity and clear striations, indicative of the printing method, were also observed. The 30 ℃ specimen presented a relatively lower definition compared to the U/T one. The loss of definition and blurring between the layers is marked in the 40 ℃ specimen and highly noticeable in the 50 ℃ sample, reflecting the substantial thermal effects on the resin (Fig. 5B, yellow arrows). The HVPP groups showed areas where the material appeared to have gathered, creating irregularities and a wavy surface texture, indicating increased reflow between the printed layers (Fig. 5C).

### Three-dimensional accuracy

**Fig. 6:**
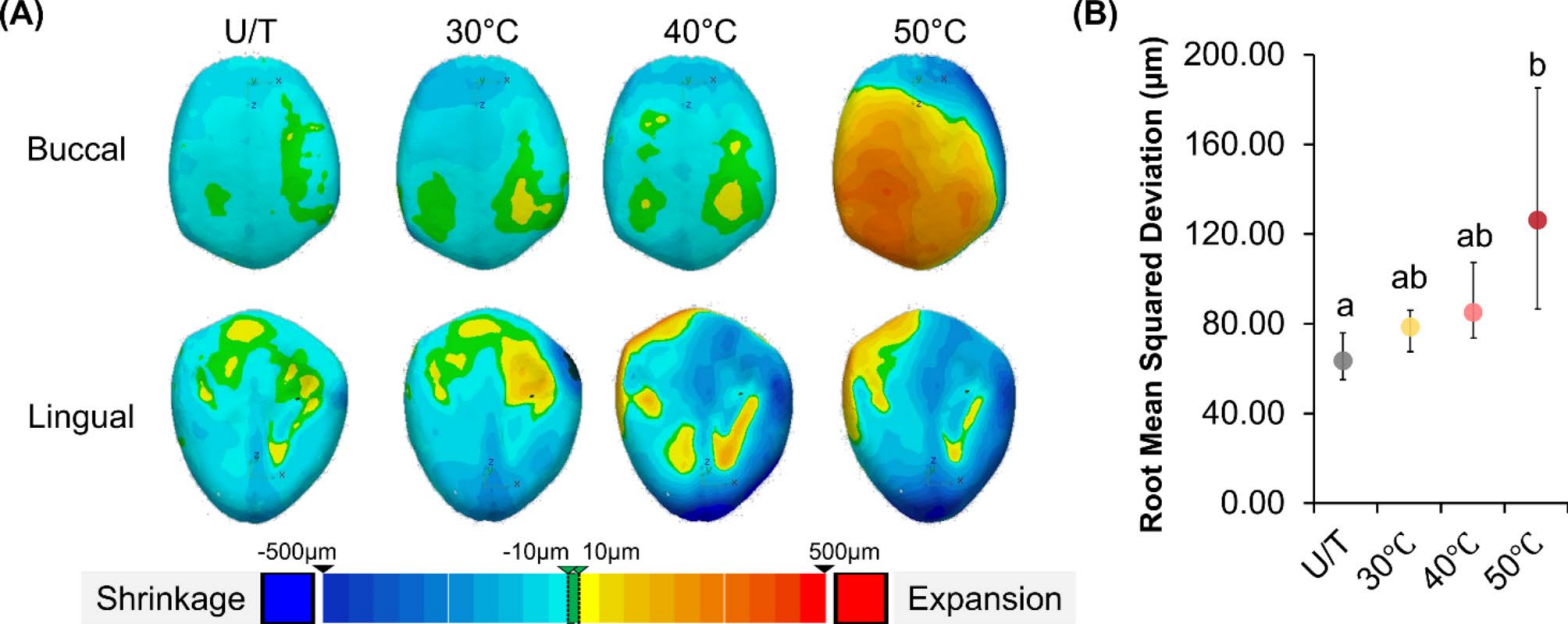
3D morphometric comparison of the accuracy of the crowns printed with high-temperature stereolithography. (**A**) Representative color map of the root mean squared deviation observed on the buccal and lingual aspects and (**B**) median RMS deviations observed per group. The different lower-case letters indicate significant differences (*P* < 0.05) between the groups, as determined by a Kruskal–Wallis multiple group comparison followed by a pairwise analysis using the Mann–Whitney U test.

Morphometric comparison of the 3D-printed crowns revealed a statistically significant difference (*P* < 0.05) in the root mean squared (RMS) values between the groups (Fig. 6B). The RMS deviations for the crowns in the 30 °C group (median: 81.10 μm, IQR: 15.83 μm) were comparable to the RMS deviations for the crowns in the U/T group (median: 64.55 μm, IQR: 18.00 μm), as confirmed by the color maps. Although the RMS deviations for the 40 °C group (median: 86.30 μm, IQR: 30.98 μm) were similar to those of the U/T group, significant positive deviations were observed on the buccal aspect of the crowns, extending lingually. In contrast, the deviation was the highest for the 50 °C group (median: 113.20 μm, IQR: 95.48 μm), suggesting a larger morphology compared to the designed CAD file (Fig. 6A). However, the RMS differences between the 30 °C, 40 °C, and 50 °C groups were not statistically significant.

## Discussion

In this study, a 3D printing resin used in clinical dentistry underwent HVPP at incremental temperatures of 30, 40, and 50 ℃. Based on the significant effect (*P <* 0.05) observed on the mechanical properties and accuracy of the 3D-printed crown, the null hypothesis was rejected.

This study explored the applicability of the HVPP technique in dentistry by evaluating its effects at different temperatures. A customized resin tank was designed to fit with a temperature controller that enabled effective control of the resin temperature during the entire printing process. The customized design adapted the use of a flat-based indium tin oxide (ITO) glass coating over the resin tank, as proposed in a previous study^20^. An earlier study reported using multiple thermocouples for direct immersion to increase the resin temperature^16^. A similar concept was adopted in the present study; however, the heating method was modified to ensure full coverage of the resin tank base. This modification ensured uniform resin heating and mitigated any inadvertent effects that could arise from changes in the printing volume and resin temperature throughout the printing process^21^.

In stereolithography (SLA)-type VPP, the separation force and resin-recoating are critical steps affecting the quality of the final print^1^. Furthermore, the viscosity of the resin is an essential factor that influences the resin and printing platform compatibility^22^. HVPP is intended to reduce the solvent; hence, higher temperatures can help in achieving favorable viscosities for printing^21^. Typically, the viscosities of commercial dental resins range from 1600 to 7300 mPa.S; however, successful SLA vat-photopolymerization is challenging above 5000 mPa.S^16,22^. The commercial resin investigated in this study had a viscosity of 2355.28 mPa.S, which was reduced by approximately 82% with an increase in temperature to 50 ℃. A further increase in temperature (> 50 ℃) resulted in microbubbles and indistinguishable streaking, possibly due to thermal degradation. Therefore, the highest HVPP temperature was limited to 50 ℃.

HVPP was investigated by first examining the UV curing process using photo-DSC. The results indicate an increased tendency for more exothermic reactions at elevated temperatures. Specifically, at 50 ℃, the presence of a high peak value signifies that the resin exhibits the most significant polymerization reactions at this temperature, further indicating a preferred HVPP temperature of 50 ℃^17^. The surface microhardness also showed a significant increase with HVPP; however, at 50 ℃, the values were relatively lower than those of the other groups. Changes in viscosity at elevated temperatures are associated with increased oxygen diffusion^23,24^. In this study, disc-shaped test samples were used to analyze the surface microhardness. The samples were printed with the disc surface oriented parallel to the z-axis; hence, oxygen diffusion was probably higher during the final curing increment. To avoid the effects of the support, the microhardness was tested on only one surface corresponding to the terminal printing layer. This could have inhibited surface cross-linking or led to monomolecular termination^23^. Considering the low depth of indentation during the Vickers microhardness testing, this effect could be limited to the tested superficial layers. Hence, the mechanical characterization was supplemented with an analysis of the flexural properties of the bulk specimens.

Given that the success of dental prostheses is critically influenced by their flexural behavior under loading, three-point bending tests were performed in accordance with the ISO^25^. The HVPP groups exhibited a significant increase in strength, elastic modulus, and modulus of resilience. Notably, HVPP at 50 ℃ showed enhanced strength, stiffness, and resilience. These findings align with previous studies reporting that HVPP at 70 ℃ enhanced the strength; however, anisotropy was also observed^16,21^. The mechanical properties of a printed object are conventionally determined by the chain length of the monomers and the cross-linking density of the polymer network formed upon UV irradiation^12,26^. Higher temperatures, which can lead to improved reaction kinetics, are likely to facilitate additional cross-linking reactions and improve the overall network structure of methacrylate-based resins^11^. This assertion is supported by the results of the photo-DSC analysis, wherein a sharp peak at 50 °C and a significant enthalpy change (424.14 mJ) are observed, indicating a possible increase in cross-linking reactions. The curing conditions at 50 °C promote the highest reaction initiation, as evidenced by a 17.58% increase in the peak heat flow compared to that at intermediate curing at 40 °C. Nonetheless, the increase in temperature is associated with higher molecular energy. It is possible that the intermediate conditions, such as 40 °C, which, although associated with an increase in molecular mobility, do not materialize into cross-linked chain termination. This may lead to the formation of a less-ordered bulk network structure that can result in suboptimal structure^23^. Conversely, at 50 °C, the reduced resin viscosity enhances interlayer adhesion and minimizes bulk-level defects. Furthermore, delayed vitrification at 50 °C enables effective stress relaxation during polymerization, resulting in a more robust and well-ordered polymer network^27^.

The printing layer topography was characterized using SEM imaging. Notable topographic changes were observed between the printed layers with increasing temperature. Boundary definitions between the layers increasingly blurred at 40 °C and 50 °C under HVPP conditions. Although the layer thickness is predefined, the thermal effects observed in this study highlight the relation between resin viscosity and temperature, which affects resin flow and layer formation^28^. At elevated temperatures, the reduced viscosity of the resin results in increased fluidity, potentially leading to non-uniform distribution and compromised layer formation accuracy. Although the gap size remains constant, heat can lead to changes in resin properties, making it harder to ensure uniform distribution before subsequent UV exposure. Similar results were reported in a previous study using confocal laser scanning microscopy, wherein higher printing temperatures lead to blurred layer boundaries^18^. This reduction in the interlayer depth was attributed to the improved adhesion between the layers. Typically, resin reflow refers to the movement and redistribution of a liquid resin during SLA printing. As each layer is cured by the laser or light source, some of the surrounding uncured resin flows back into the just-printed area^29^. In this study, a higher tendency for reflow between the layers was observed (Fig. 4C). The resin viscosity primarily affects the reflow during printing; hence, these changes can be attributed to the same factor.

Typically, low-viscosity resins are preferred owing to their ability to replenish each layer during printing and their ease of handling. However, they may result in a high shrinkage and negatively impact the quality of the finished products^30^. Given the sensitivity of resins to volume shrinkage, the morphological dimensions of the 3D-printed interim prosthesis were examined^31^. In this study, the HVPP groups showed significantly higher RMS values, agreeing with previous studies reporting a maximum deviation of 85 μm^18^. Furthermore, our results showed that HVPP at 50 ℃ afforded higher deviations, with the values being within the previously reported 150 μm threshold^32,33^. Color map analysis of the crown structure revealed that the volume of the printed crowns was larger (yellow-red spectrum).

In this study, crowns were printed occluso-gingivally, with a median RMS value of 131 μm, which is within the clinical threshold of 150 μm. However, the 50 °C group showed high variability. The improved resin flow at higher temperatures reduced viscosity but may have caused reflow between layers^29^. During post-curing, this reflow likely increased the overall volume of the crown. Therefore, better printer calibration is necessary in high-temperature printing to reduce these variations. This tendency for the preheated resin to resist shrinkage or increase in volume has also been observed in previous studies and attributed to the delay in resin vitrification^34^. Additionally, heating of the resin can contribute to relieving emerging shrinkage stress through improved viscous flow and polymer chain relaxation^35^. In summary, the enhanced initial compliance and extended stress relief in the preheated resin help mitigate shrinkage force formation^31^.

In this study, we investigated the applicability of HVPP for the development of a clinically relevant dental resin. Our results revealed the positive influence of HVPP on the mechanical properties of the 3D-printed parts. However, this study was limited to a single-resin formulation, and the effects of the printing speed and orientation were not factored in, although these factors could have potential effects, as reported previously^21^. A further limitation was the absence of dynamic condition testing, such as fatigue loading using a chewing simulator. In general, the study groups demonstrated a favorable degree of double bond conversion (Supporting Table S2), supporting good biocompatibility. However, the cross-linking structure of the resin is vulnerable to mechanical and hydrothermal fatigue. Therefore, future studies should prioritize investigating the physical, mechanical, and biological properties of HVPP resins following exposure to fatigue. These results lay the groundwork for future research, particularly for exploring the influence of HVPP on factors such as diluent concentration in the resin, printing time, surface smoothness, and optimization of CAD design. Further research focusing on hydrothermal and mechanical aging would help detail the effectiveness of cross-linking and its contribution to prosthesis longevity.

## Conclusion

In this study, we compared and presented the effect of the 3D printing temperature on the properties of a 3D-printed acrylic resin. Within the limitations of this study, it can be concluded that during HVPP, the properties of acrylate-type resins are affected by temperature. Heat treatment of the resin in combination with the 3D printing protocol improved the mechanical properties. Thus, these findings present a promising basis that could aid in the development of future standardized protocols for solvent-free acrylic materials for resin-based dental prosthesis.

## Methods

### Custom resin tank fabrication

**Fig. 7:**
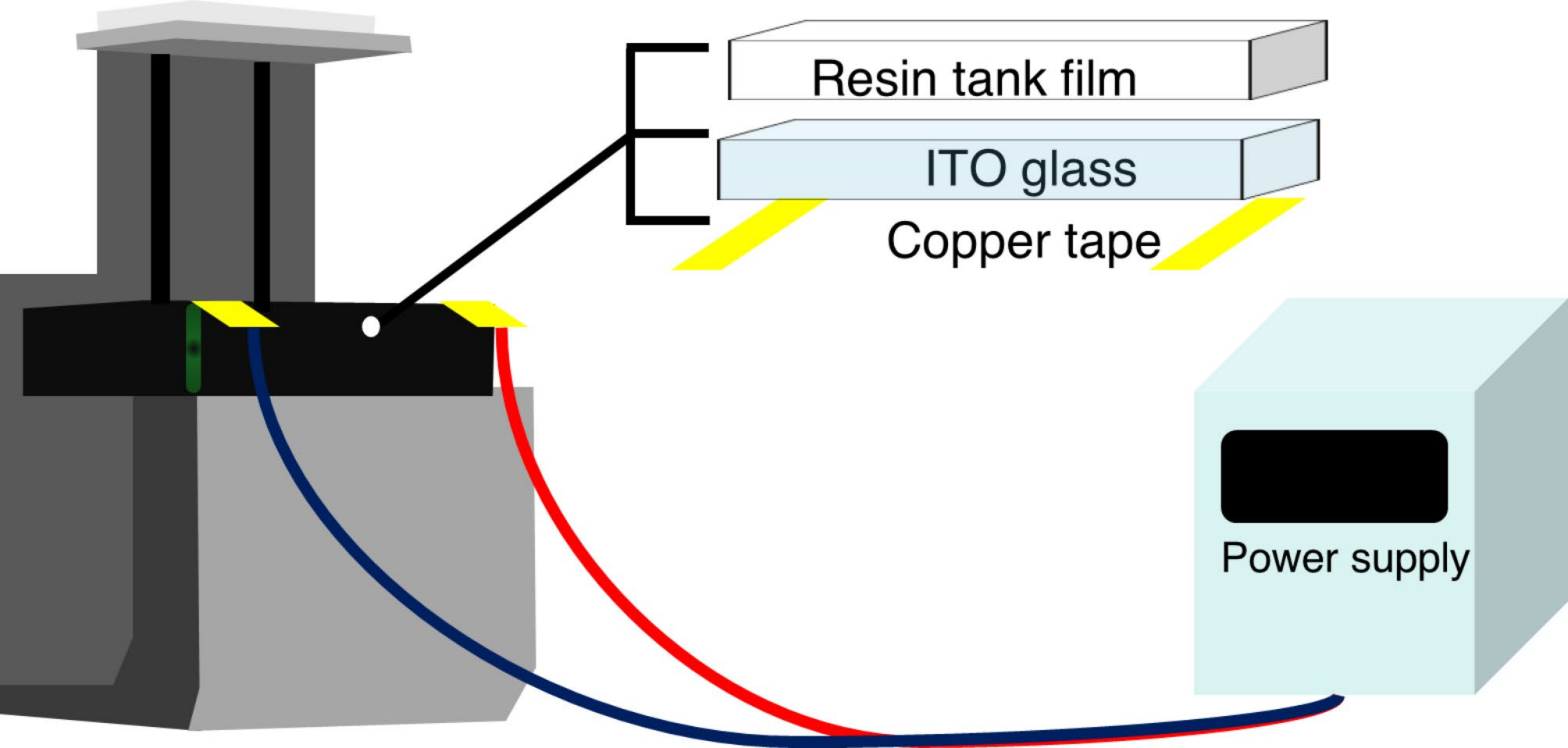
Schematic of the custom resin tank designed for temperature control.

A commercial SLA 3D printer (A1, Shindo, Korea) was modified to implement a hot-lithography system that directly transfers heat to the resin beneath the resin tank (Fig. 7). This modification was based on a previous report by Kuhnt et al.^20^ Briefly, the commercial printer had a vat configured with a flat-based ITO-coated glass. A copper tape was glued to the bottom of the main ITO-coated glass for heat conduction. The copper tape was extended to the outside of the 3D printer. The heating system operates as the extended copper tape is connected to the power supply.

### 3D printing and specimen fabrication

**Fig. 8:**
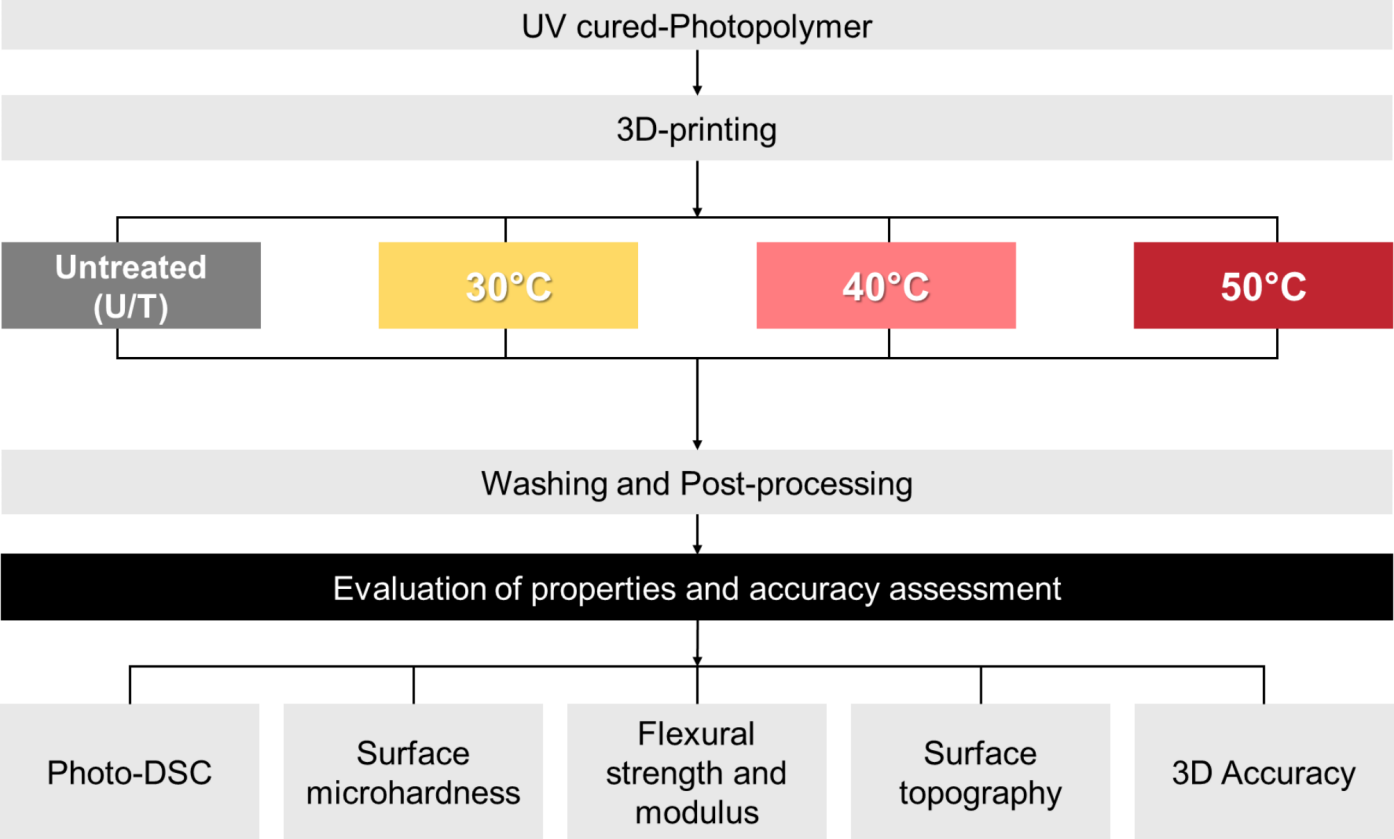
Flowchart presenting the study design and experimental workflow.

A commercial resin (TeraHarz TC-80DP A2; Graphy, Korea) clinically indicated for crown and bridge prosthesis was used as the 3D printing material for the vat photopolymerization. The resin was filled into the tank and pre-heated at varying temperatures according to the different test groups (30 ℃, 40 ℃, and 50 ℃). The temperature was monitored (K-type thermometer Center 306, Center Technology, Taipei, Taiwan) and kept stable throughout the printing process. After the 3D printing process, the specimens were washed with ethyl alcohol 80% for 10 min and dried by light blocking. The post-curing process was performed for 20 min under a maximum light intensity of 2,200 mW/cm^2^ at 300–550 nm (LC-3D Print Box, Nextdent by 3D Systems, Netherlands). The HVPP samples were grouped according to their temperatures during printing. The samples printed with no heat pre-treatment (Untreated; U/T) had an average temperature of 25.29 ± 0.3 ℃ after loading into the resin tank. Samples for each test were prepared in accordance with established guidelines and methods reported in previous studies. Sample sizes and dimensions were chosen based on relevant standards and earlier research to ensure consistency and reliability. The experimental workflow is presented in Fig. 8.

### Viscosity

The viscosity of the photopolymer resin for 3D printing was determined using a rheometer (Anton Paar, MCR 102e with parallel-plate 25 mm in diameter, Austria) with a parallel plate 22 mm in diameter with a 25-µm gap from the base. The flow curves were drawn with fixed shear rates of 5 s-1 at 20–50 ℃.

### Photo-differential scanning calorimetry (PHOTO-DSC)

Photo-DSC was performed on a Netzsch DSC 204 F1 Phoenix^®^ to analyze the key parameters of the photopolymerization process, such as reaction enthalpy^17^. Before each experiment, the DSC chamber was purged with N_2_ for 30 min at a rate of 20 mL/min prior to irradiation to remove the oxygen from the samples. All tests were performed in isothermal conditions at different temperatures, at 30, 40, and 50 ℃. The resins (10–15 mg) were irradiated twice with filtered UV light (320–500 nm) using an Exfo OmniCure™ Series 2000 broadband Hg lamp. The light intensity was set at ~ 60 mW/cm^2^ on the surface of the sample. The heat flow of the polymerization reaction was recorded as a function of time, and the temperature was maintained at a constant value. All measurements were performed in triplicate, exhibiting satisfactory reproducibility.

### Surface microhardness

The Vickers hardness (VH) of the 3D-printed specimens was measured using a microhardness tester (MMT-X, Matsuzawa, Japan). For the surface hardness measurement, a force of 300 gf with a dwell time of 25 s was loaded using an indenter in the form of a diamond pyramid at 136° on top of the layer surface of the 3D-printed specimens. Each test was randomly performed in triplicate for each specimen. The results are reported as the average of 10 specimens for each group^9^.

### Flexural strength, modulus, and resilience

The flexural strength, flexural modulus, and resilience were tested according to the International Standard ISO 10,477. Ten specimens (25 × 2 × 2 mm^3^) were immersed in distilled water and stored at 37 ℃ for 24 h. Thereafter, all the specimens were loaded to fracture using a universal testing machine (EZ-LX, Shimadzu, Kyoto, Japan) with a span length of 20 mm and a crosshead speed of 1 mm/min. The flexural strength ($\:\sigma\:)$ and flexural modulus (E) were measured in MPa using Eqs. (1) and (2).

1 $$\:\sigma\:=\frac{3Fl}{{2bh}^{2}}$$

2 $$\:E=\frac{{{F}_{1}l}^{3}}{{4bh}^{3}d}$$

where F (N) is the maximum load applied to the specimen, l is the distance between the supports (mm), and b and h are the width and height of the specimen (mm), respectively. *F*_1_ is the load at a point in the straight-line portion of the load/displacement curve, and *d* is the deflection at load *P* (mm).

After determining the flexural strength and modulus, the modulus of resilience was calculated using Eq. (3).

3 $$\:R=\frac{{S}^{2}}{2E}$$

where R, S, and E are the moduli of the resilience, flexural strength, and flexural modulus, respectively. The results are reported as the average of 10 specimens for each group.

### Printing layer topography

SEM images were obtained to analyze the 3D printing build direction surface morphology and the boundaries between the printed layers. Samples from each group were randomly selected and sputter-coated with gold for 60 s. Their surface was observed via FE-SEM (JEOL-7800 F, JEOL, Tokyo, Japan) at an operating voltage of 5 kV with ×90 and ×250 magnification.

### Three-dimensional accuracy of dental crowns

The 3D-printed canine teeth in each group were digitized using a desktop scanner (T500; Medit Corp, Seoul, South Korea) to obtain a reference standard tessellation language (STL) dataset (*n* = 5)^18^. The reference STL file was used in the CAD/CAM software for 3D printing. The reference and scan files were loaded into a 3D inspector program (Geomagic Control X, 3D Systems, Rock Hill, USA). The scanned file was initially aligned based on a reference file to match its orientation, followed by a best-fit alignment between the meshes. RMS values were used to measure the 3D discrepancies between the reference file and the specimen scan file. The values obtained from each specimen comparison were analyzed to evaluate the dimensional accuracy of HVPP at different temperatures. The magnitude and deviation patterns of the 3D-printed specimen relative to those of the reference specimen were visualized using color-coded deviation maps.

### Statistical analysis

Statistical analysis was performed using IBM SPSS Statistics v27.0 (IBM Corp., Armonk, NY). One-way ANOVA with Tukey’s post hoc test was used to evaluate the surface microhardness, flexural strength, modulus, and resilience. The Kruskal–Wallis test was used to assess the differences in the 3D accuracy of the crowns among the groups, followed by Mann–Whitney U tests for post hoc analysis. The Bonferroni method was used to adjust for multiple comparisons, with significance set at *P* < 0.05.

## Electronic supplementary material

Below is the link to the electronic supplementary material.

Supplementary Material 1

## Data Availability

The data that support the figures and tables within this paper are presented in the main text. All other additional data are available from the corresponding author upon request.
