# Supplementary material for: Influence of heat-assisted vat photopolymerization on the physical and mechanical characteristics of dental 3D printing resins
Source: Sci Rep. 2025 Jan 11;15:1710. doi: 10.1038/s41598-025-85529-7 (PMC11724836; doi:10.1038/s41598-025-85529-7)
Supplement: Supplementary file 1 — Supplementary Material 1 [file 41598_2025_85529_MOESM1_ESM.docx]

Supporting information

Influence of Heat-Assisted Vat Photopolymerization on the Physical and Mechanical Characteristics of Dental 3D Printing Resins

Jung-Hwa Lim^1^, Seung-Ho Shin^1^, Young-Eun Jung^2^, Hongseok An^3^, Jong-Eun Kim^1*^

^1^ Department of Prosthodontics, Yonsei University College of Dentistry, Yonsei-ro 50-1, Seodaemun-gu, Seoul 03722, Republic of Korea

^2^ Department of Orthodontics, New York University College of Dentistry, 345 E 24th St, New York, NY 10010, USA

^3^ Department of Oral Rehabilitation and Biosciences, Oregon Health & Science University School of Dentistry, 2730 S Moody Ave, Portland, Oregon 97201, USA

*Email of the corresponding author: gomyou@yuhs.ac

**Table S1**. Effects of high temperature stereolithography at different temperature on properties dental 3D printing resin. All values presented in mean±SD

| Group | Flexural strength (MPa) | Flexural Modulus (MPa) | Modulus of resilience (MJ/mm^3^) |
| --- | --- | --- | --- |
| U/T | 59.24 ± 5.52^a^ | 2526.48 ± 127.58 ^a^ | 0.70 ± 0.11 ^a^ |
| 30℃ | 75.56 ± 8.01^b^ | 2824.08 ± 241.96 ^b^ | 1.03 ± 0.25 ^b^ |
| 40℃ | 70.52 ± 10.27^b^ | 2700.57 ± 119.74 ^a^ | 0.94 ± 0.28 ^a^ |
| 50℃ | 90.33 ± 11.56^c^ | 3618.88 ± 339.74 ^b^ | 1.14 ± 0.25 ^c^ |

**Double bond conversion analysis**

The FTIR spectra for the unpolymerized and polymerized urethane-based acrylate samples were recorded using a Fourier-transform infrared spectrometer (Nicolet is10, Thermo Fisher Scientific, USA). The spectra were collected in the range of 4000–400 cm⁻¹ with a resolution of 4 cm⁻¹, and the absorbance values were extracted for analysis.

To quantify the degree of polymerization, the relative changes in absorbance of the double bond (C=C) peak were analyzed. The C=C peak reflecting the unreacted monomer and the C-H stretching serving as the normalization reference and the double bond conversion (DBC) was calculated as:

$$\text{DBC }\left( \text{\%} \right)=\left( 1-\frac{{Area}_{C=C, polymerized}\text{ }{/Area}_{C-H, polymerized}}{{Area}_{C=C, unpolymerized}\text{ }{/Area}_{C-H,unpolymerized}} \right)\times100$$

Where,

Area_C=C_ is the area under the C=C peak (1630–1640 cm⁻¹) and Area_C-H_ is the area under the C-H stretching region (2800–3000 cm⁻¹).

**Table S2**. Double bond conversion percentage

| Group | Double bond conversion (%) |
| --- | --- |
| U/T | 97.04% |
| 30°C | 99.36% |
| 40°C | 99.46% |
| 50°C | 98.13% |
